# Supplementary material for: Machine Learning Accelerates Crystallization for Structure Determination
Source: Angew Chem Int Ed Engl. 2026 May 4;65(25):e1218503. doi: 10.1002/anie.1218503 (PMC13266958; doi:10.1002/anie.1218503)

checkCIF/PLATON report

Structure factors have been supplied for datablock(s) lcz075

THIS REPORT IS FOR GUIDANCE ONLY. IF USED AS PART OF A REVIEW PROCEDURE FOR PUBLICATION, IT SHOULD NOT REPLACE THE EXPERTISE OF AN EXPERIENCED CRYSTALLOGRAPHIC REFEREE.

No syntax errors found. CIF dictionary Interpreting this report

Datablock: lcz075

Bond precision: C-C = 0.0160 A Wavelength=1.54184

Cell:	a=7.9092(2)	b=9.6128(2)	c=19.9883(4)
	alpha=85.900(2)	beta=88.992(2)	gamma=89.601(2)
Temperature:	100 K		

	Calculated	Reported
Volume	1515.55(6)	1515.55(6)
Space group	P -1	P -1
Hall group	-P 1	-P 1
Moiety formula	C15 H3 Ag3 F18 N6, C8 H9 N O	C15 H3 Ag3 F18 N6, C8 H9 N O
Sum formula	C23 H12 Ag3 F18 N7 O	C23 H12 Ag3 F18 N7 O
Mr	1068.01	1068.01
Dx,g cm-3	2.340	2.340
Z	2	2
Mu (mm-1)	16.780	16.780
F000	1020.0	1020.0
F000'	1025.01	
h,k,lmax	10,12,25	10,12,25
Nref	6516	6191
Tmin,Tmax	0.087,0.187	0.350,1.000
Tmin'	0.014	

Correction method= # Reported T Limits: Tmin=0.350 Tmax=1.000
AbsCorr = MULTI-SCAN

Data completeness= 0.950 Theta(max)= 78.568

R(reflections)= 0.0713(5431) wR2(reflections)= 0.2014(6191)

S = 1.050 Npar= 471

The following ALERTS were generated. Each ALERT has the format
test-name_ALERT_alert-type_alert-level.
Click on the hyperlinks for more details of the test.

Alert level C

```
PLAT213_ALERT_2_C Atom F011           has ADP max/min Ratio .....        3.9 prolat
PLAT213_ALERT_2_C Atom F017           has ADP max/min Ratio .....        3.9 prolat
PLAT213_ALERT_2_C Atom F01D           has ADP max/min Ratio .....        3.8 prolat
PLAT220_ALERT_2_C NonSolvent   Resd 1  F   Ueq(max)/Ueq(min) Range        4.7 Ratio
PLAT342_ALERT_3_C Low Bond Precision on  C-C Bonds ..............    0.01595 Ang.
PLAT906_ALERT_3_C Large K Value in the Analysis of Variance ......      3.839 Check
PLAT911_ALERT_3_C Missing FCF Refl Between Thmin & STh/L=    0.600         36 Report
                 9  0  0,  -1  3  0,  -2 11  2,   3 -7  3,   4 -4  4,  -2 -3  7,
                 9  2  7,   1  0  8,   1  4  8,  -1 11  8,  -2  3  9,   0 -1 10,
                -2  2 10,  -1 10 10,   4 -3 11,  -7  2 11,  -8 -1 12,   6 -1 12,
                -8  0 12,  -7  0 12,  -8  1 12,  -8  2 12,  -7  0 13,  -7  1 13,
                -7  2 13,  -7  0 14,  -7  1 14,  -6  6 14,  -7 -1 15,  -7  0 15,
                    (    6 More Missing: see the .ckf listing file)
PLAT934_ALERT_3_C Number of (Iobs-Icalc)/Sigma(W) > 10 Outliers ..          1 Check
                 1  3  2,
PLAT971_ALERT_2_C Check Calcd Resid. Dens.  1.03Ang From Ag01          2.23 eA-3
PLAT971_ALERT_2_C Check Calcd Resid. Dens.  1.20Ang From Ag02          1.97 eA-3
PLAT971_ALERT_2_C Check Calcd Resid. Dens.  1.17Ang From Ag03          1.96 eA-3
PLAT971_ALERT_2_C Check Calcd Resid. Dens.  1.17Ang From F01D          1.92 eA-3
PLAT971_ALERT_2_C Check Calcd Resid. Dens.  1.14Ang From Ag02          1.80 eA-3
PLAT971_ALERT_2_C Check Calcd Resid. Dens.  1.01Ang From F01D          1.70 eA-3
PLAT971_ALERT_2_C Check Calcd Resid. Dens.  1.33Ang From F011          1.68 eA-3
PLAT971_ALERT_2_C Check Calcd Resid. Dens.  1.12Ang From Ag01          1.55 eA-3
PLAT972_ALERT_2_C Check Calcd Resid. Dens.  0.75Ang From Ag03         -1.63 eA-3
PLAT977_ALERT_2_C Check Negative Difference Density on H00B      .      -0.46 eA-3
```

Alert level G

```
PLAT007_ALERT_5_G Number of Unrefined Donor-H Atoms .............          2 Report
              H00A  H00B
PLAT083_ALERT_2_G SHELXL Second Parameter in WGHT  Unusually Large      38.26 Why ?
PLAT154_ALERT_1_G The s.u.'s on the Cell Angles are Equal ..(Note)      0.002 Degree
PLAT242_ALERT_2_G Low    'MainMol' Ueq as Compared to Neighbors of       C00W Check
PLAT242_ALERT_2_G Low    'MainMol' Ueq as Compared to Neighbors of       C012 Check
PLAT242_ALERT_2_G Low    'MainMol' Ueq as Compared to Neighbors of       C016 Check
PLAT242_ALERT_2_G Low    'MainMol' Ueq as Compared to Neighbors of       C01A Check
PLAT242_ALERT_2_G Low    'MainMol' Ueq as Compared to Neighbors of       C01E Check
PLAT434_ALERT_2_G Short Inter HL..HL Contact F007     ..F009     .       2.79 Ang.
                                         -x,1-y,1-z  =     2_566 Check
PLAT434_ALERT_2_G Short Inter HL..HL Contact F00K     ..F011     .       2.80 Ang.
                                            x,1+y,z  =     1_565 Check
PLAT720_ALERT_4_G Number of Unusual/Non-Standard Labels .........         64 Note
```

```
              Ag01    Ag02    Ag03    F004    F005    F006    F007    F008
              F009    F00A    F00B    F00C    N00D    F00E    F00F    N00G
              O00H    N00I    F00J    F00K    N00L    F00M    F00N    N00O
              N00P    N00Q    H00A    H00B    C00R    C00S    C00T    C00U
              H00U    C00V    C00W    C00X    H00X    C00Y    H00Y    C00Z
              H00Z    C010    F011    C012    C013    C014    C015    C016
              F017    C018    H018    C019    C01A    C01B    C01C    H01C
              F01D    C01E    C01F    H01A    H01B    H01D    C01G    H01G
PLAT883_ALERT_1_G Absent Datum for _atom_sites_solution_primary ..     Please Do !
PLAT910_ALERT_3_G Missing FCF Reflection(s) Below Theta(Min)[Deg]=       4.44 Note
                0  0  1,
PLAT912_ALERT_4_G Missing # of FCF Reflections Above STh/L=  0.600        288 Note
PLAT941_ALERT_3_G Average HKL Measurement Multiplicity ..........         2.6 Low
PLAT969_ALERT_5_G The 'Henn et al.' R-Factor-gap value ..........       3.916 Note
              Predicted wR2: Based on SigI**2  5.14 or SHELX Weight 19.18
PLAT978_ALERT_2_G Number C-C Bonds with Positive Residual Density.          0 Info
```

```
   0 ALERT level A = Most likely a serious problem - resolve or explain
   0 ALERT level B = A potentially serious problem, consider carefully
  18 ALERT level C = Check. Ensure it is not caused by an omission or oversight
  17 ALERT level G = General information/check it is not something unexpected

   2 ALERT type 1 CIF construction/syntax error, inconsistent or missing data
  23 ALERT type 2 Indicator that the structure model may be wrong or deficient
   6 ALERT type 3 Indicator that the structure quality may be low
   2 ALERT type 4 Improvement, methodology, query or suggestion
   2 ALERT type 5 Informative message, check
```

Validation response form

Please find below a validation response form (VRF) that can be filled in and pasted into your CIF.

```
# start Validation Reply Form
_vrf_PLAT213_lcz075
;
PROBLEM: Atom F011            has ADP max/min Ratio .....        3.9 prolat
RESPONSE: ...
;
_vrf_PLAT220_lcz075
;
PROBLEM: NonSolvent   Resd 1  F   Ueq(max)/Ueq(min) Range        4.7 Ratio
RESPONSE: ...
;
_vrf_PLAT342_lcz075
;
PROBLEM: Low Bond Precision on  C-C Bonds ..............    0.01595 Ang.
RESPONSE: ...
;
_vrf_PLAT906_lcz075
;
PROBLEM: Large K Value in the Analysis of Variance ......      3.839 Check
RESPONSE: ...
;
_vrf_PLAT911_lcz075
;
```

```
PROBLEM: Missing FCF Refl Between Thmin & STh/L=    0.600         36 Report
RESPONSE: ...
;
_vrf_PLAT934_lcz075
;
PROBLEM: Number of (Iobs-Icalc)/Sigma(W) > 10 Outliers ..          1 Check
RESPONSE: ...
;
_vrf_PLAT971_lcz075
;
PROBLEM: Check Calcd Resid. Dens.  1.03Ang From Ag01           2.23 eA-3
RESPONSE: ...
;
_vrf_PLAT972_lcz075
;
PROBLEM: Check Calcd Resid. Dens.  0.75Ang From Ag03          -1.63 eA-3
RESPONSE: ...
;
_vrf_PLAT977_lcz075
;
PROBLEM: Check Negative Difference Density on H00B      .      -0.46 eA-3
RESPONSE: ...
;
# end Validation Reply Form
```

It is advisable to attempt to resolve as many as possible of the alerts in all categories. Often the minor alerts point to easily fixed oversights, errors and omissions in your CIF or refinement strategy, so attention to these fine details can be worthwhile. It is up to the individual to critically assess their own results and, if necessary, seek expert advice.

PLATON version of 17/09/2025; check.def file version of 17/09/2025

---

duplicate check

No duplication found

Datablock lcz075 - ellipsoid plot

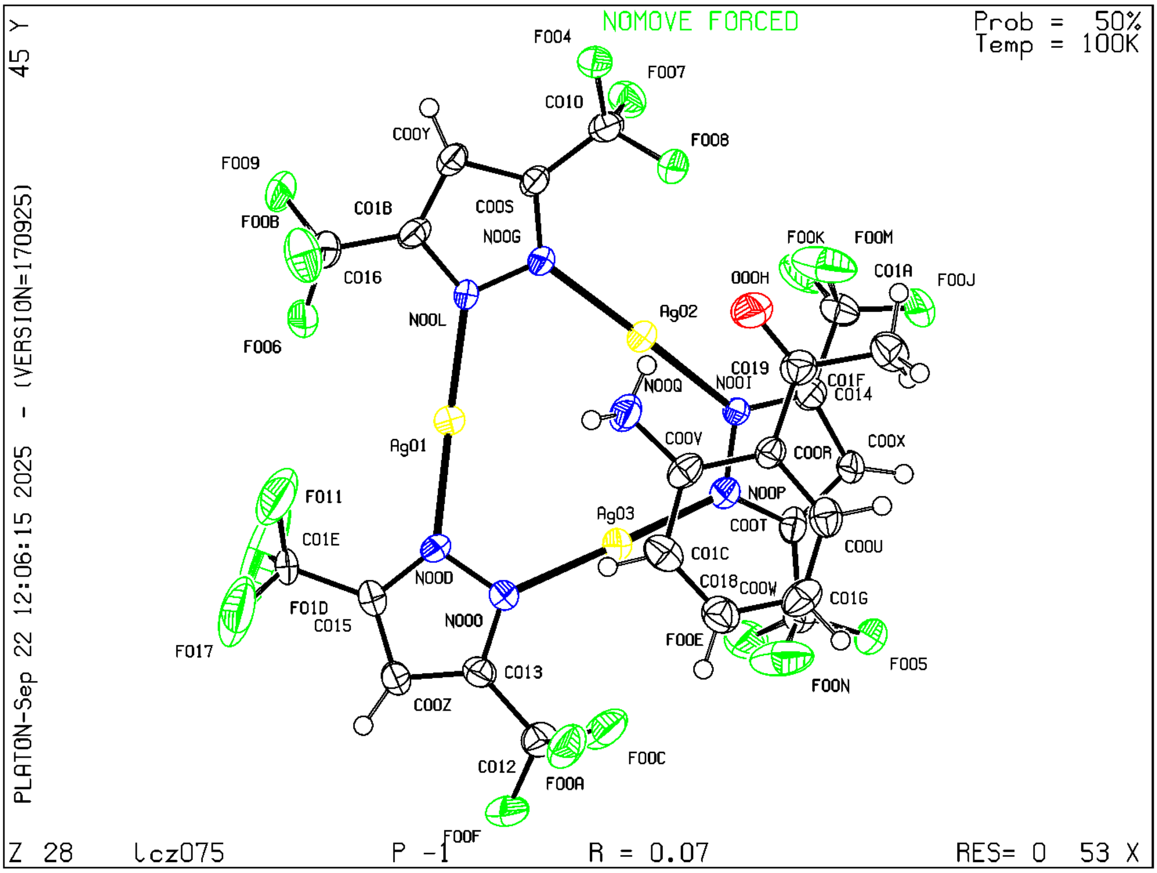

Supplement: Supplementary file 2 — Supporting File 2: anie72462‐sup‐0002‐DataFile.zip. [file ANIE-65-e1218503-s002.zip › supplementary_materials_crystallographic_data submit.zip!/cif_and_checkcif(1-114)/Ag3Pz3_75/Ag3Pz3_75_checkcif.pdf]
